# The effect of intravenous maropitant on blood pressure in healthy awake and anesthetized dogs

**DOI:** 10.1371/journal.pone.0229736

**Published:** 2020-02-27

**Authors:** Ting-Ting Chi, Bonnie L. Hay Kraus

**Affiliations:** Department of Veterinary Clinical Sciences, Iowa State University College of Veterinary Medicine, Ames, Iowa, United States of America; The University of Manchester, UNITED KINGDOM

## Abstract

**Objective:**

To evaluate the effects of intravenous maropitant on arterial blood pressure in healthy dogs while awake and under general anesthesia.

**Design:**

Experimental crossover study.

**Animals:**

Eight healthy adult Beagle dogs.

**Procedure:**

All dogs received maropitant (1 mg kg^-1^) intravenously under the following conditions: 1) awake with non-invasive blood pressure monitoring (AwNIBP), 2) awake with invasive blood pressure monitoring (AwIBP), 3) premedication with acepromazine (0.005 mg kg^-1^) and butorphanol (0.2 mg kg^-1^) intramuscularly followed by propofol induction and isoflurane anesthesia (GaAB), and 4) premedication with dexmedetomidine (0.005 mg kg^-1^) and butorphanol (0.2 mg kg^-1^) intramuscularly followed by propofol induction and isoflurane anesthesia (GaDB). Heart rate (HR), systolic (SAP), diastolic (DAP), and mean blood pressures (MAP) were recorded before injection of maropitant (baseline), during the first 60 seconds of injection, during the second 60 seconds of injection, at the completion of injection and every 2 minutes post injection for 18 minutes. The data were compared over time using a Generalized Linear Model with mixed effects and then with simple effect comparison with Bonferroni adjustments (*p* <0.05).

**Results:**

There were significant decreases from baseline in SAP in the GaAB group (*p* < 0.01) and in MAP and DAP in the AwIBP and GaAB (*p* < 0.001) groups during injection. A significant decrease in SAP (*p* < 0.05), DAP (*p* < 0.05), and MAP (*p* < 0.05) occurred at 16 minutes post injection in GaDB group. There was also a significant increase in HR in the AwIBP group (*p* < 0.01) during injection. Clinically significant hypotension occurred in the GaAB group with a mean MAP at 54 ± 6 mmHg during injection.

**Conclusion:**

Intravenous maropitant administration significantly decreases arterial blood pressure during inhalant anesthesia. Patients premedicated with acepromazine prior to isoflurane anesthesia may develop clinically significant hypotension.

## Introduction

Maropitant is a neurokinin (NK-1) receptor antagonist that inhibits binding of substance P (SP) in the chemoreceptor trigger zone (CTZ) and the vomiting center (VC), thereby inhibiting emesis in dogs and cats [1]. NK-1 receptors are found in the central nervous system and peripheral tissues and are involved in pain transmission, vasodilation, inflammatory response modulation, and sensory neuronal transmission [1,2].

Maropitant is effective in decreasing opioid and alpha-2 agonist induced vomiting and nausea when administered subcutaneously or orally prior to premedication, thereby decreasing patient discomfort and risk of peri-anesthetic aspiration pneumonia [2–7]. Dogs receiving maropitant experience improved quality of anesthetic recovery and shortened time to return to postoperative feeding, which helps mitigate the negative energy balance associated with surgery and anesthesia [8]. Maropitant may also have a role in providing adjunct analgesia for visceral pain as it has been shown to decrease the anesthetic inhalant requirement during ovariohysterectomy in dogs and cats [9–14]. Due to the multiple benefits in anesthetic and surgical patients, maropitant is frequently incorporated into anesthetic protocols in canine and feline patients. Peri-anesthetic injectable maropitant is often administered via the subcutaneous (SC) route. The disadvantages of SC administration include pain on injection and the relatively long onset of action of one hour for prevention of vomiting and signs of nausea [15]. Intravenous (IV) administration was added to the USA label in 2016 which decreases the time for onset of action and avoids painful SC injection.

Studies by *Boscan et al* and *Alvillar et al* observed a transient decrease in mean arterial blood pressure for approximately 10 minutes when maropitant was administered IV in healthy dogs under general anesthesia [14,16]. These studies did not quantify the statistical significance of the observed changes in mean arterial blood pressure. To the author’s knowledge, there are no published studies or proprietary literature regarding the effect of IV administration of maropitant on arterial blood pressure in either awake or anesthetized dogs.

The primary goal of this study was to document the effect of IV administration of maropitant on arterial blood pressure in a controlled setting with healthy awake and anesthetized dogs. The effects of maropitant were evaluated with different premedication drugs (acepromazine, dexmedetomidine, and butorphanol) which are commonly used in clinical veterinary anesthesia [17]. Acepromazine is an *α*_1_-adrenergic receptor antagonist that causes a dose dependent decrease in mean arterial blood pressure due to vasodilation [18]. Dexmedetomidine is *α*_2_-adrenergic receptor agonist that increases systemic arterial blood pressure by *α*_2_- adrenergic receptor-mediated constriction of the vascular smooth muscles of arterial vessels [18]. Butorphanol is a κagonist-μantagonist opioid that provides sedation and mild analgesia with minimal cardiovascular effects [19]. We hypothesized that maropitant would cause a transient decrease in blood pressure with intravenous administration in heathy awake and anesthetized dogs.

## Materials and methods

This study was approved by the Iowa State University Institutional Animal Care and Use Committee (protocol number 2-18-8702-K). This study was carried out in accordance with the recommendations in the Guide for the Care and Use of Laboratory Animals of the National Institutes of Health, and all efforts were made to minimize patient distress and suffering.

Eight research spayed female beagles from Iowa State University Laboratory Animal Resource (ISU LAR) were studied in a crossover design. All dogs were 2 years of age and the mean body weight was 8.7 ± 0.8 kg. The dogs were assigned an ASA (American Society of Anesthesiologists) physical status of 1 (healthy, no physical abnormalities) based on physical examination. Dogs were fasted for 12 hours prior to anesthesia but had free access to water up until the time of the study. The invasive blood pressure transducer was calibrated against a mercury manometer prior to the start of each experiment. The dogs underwent 4 treatment protocols each preceded by a 72 hour washout period: 1) awake with indirect (oscillometric) blood pressure monitoring, 2) awake with direct blood pressure monitoring, 3) general anesthesia with direct blood pressure monitoring after premedication with acepromazine/butorphanol, and 4) general anesthesia with direct blood pressure monitoring after premedication with dexmedetomidine/butorphanol. General anesthesia was induced with propofol and followed by isoflurane inhalant anesthesia. Each dog served as its own control.

### Awake protocols

In the awake protocol with non-invasive blood pressure monitoring (AwNIBP), blood pressure was measured with an oscillometric blood pressure monitor (Cardell^®^ 9402). Dogs were placed in right lateral recumbency and allowed 5 minutes acclimation time. Lidocaine (2.5 mg) (Lidocaine 2% injection, VETone^®^) subcutaneous (SC) was injected at the cephalic catheter site with an insulin syringe (U-100 Insulin Syringes with Ultra-Fine^™^ needle, BD^®^) to prevent discomfort associated with IV catheter placement. A 20-gauge IV catheter (Surflo^®^, Terumo^®^) was placed in the cephalic vein. Maropitant (1 mg kg^-1^) (Cerenia^®^, Zoetis^®^) IV was administered over 2 minutes using hand injection with a timer. The administration time was determined according to the manufacturer package insert, which states that maropitant should be administered intravenously over 1–2 minutes. The blood pressure cuff was sized at 40% of limb circumference and placed on the pelvic limb proximal to the tarsus. Heart rate (HR) and oscillometric pressure readings of systolic (SAP), diastolic (DAP), and mean arterial pressure (MAP) were obtained prior to injection (baseline), at completion of injection (Tc), and every 10 minutes for 20 minutes after injection. Three readings were obtained at each recording time point and averaged.

In the awake group with invasive blood pressure (AwIBP), dogs were placed in right lateral recumbency and allowed 5 minutes acclimation time. Lidocaine was injected SC at the cephalic (2.5 mg) and dorsal pedal (2.5 mg) catheter sites with an insulin syringe (U-100 Insulin Syringes with Ultra-Fine^™^ needle, BD^®^). A 20-gauge IV catheter was placed in the cephalic vein. Then, a 22-gauge catheter was placed in the dorsal pedal artery for invasive blood pressure monitoring (Mindray, Datascope Spectrum^®^). The dorsal pedal artery arterial catheter was attached to a low-compliance pressure tubing that connected to an electronic pressure transducer positioned and zeroed at the level of the sternal manubrium. Maropitant (1 mg kg^-1^ IV) was administered over 2 minutes using hand injection with a timer. Heart rate, SAP, DAP and MAP were recorded before injection (baseline), at the start of injection (T_0_), during the first 60 seconds of injection (T_a_), during the second 60 seconds of injection (T_b_), at completion of injection (T_c_) and every 2 minutes post injection (T_-2_) for 18 minutes (T_-18_).

### General anesthesia protocols

Dogs were premedicated with acepromazine (0.005 mg kg^-1^) (Acepromazine injection, VETone^®^) and butorphanol (0.2 mg kg^-1^) (Torbugesic^®^-SA, Zoetis^®^) (GaAB group) or dexmedetomidine (0.005 mg kg^-1^) (Dexdomitor^®^, Zoetis^®^) and butorphanol (0.2 mg kg^-1^) (Torbugesic^®^-SA, Zoetis^®^) (GaDB group) intramuscularly (IM) in the lumbar epaxial muscles. The premedication drug doses were chosen based on doses commonly used at this institution for clinical patients undergoing anesthesia. Fifteen minutes after premedication, a 20-gauge IV cephalic catheter was placed after local block with lidocaine (2.5 mg SC) over the catheter site with an insulin syringe (U-100 Insulin Syringes with Ultra-Fine^™^ needle, BD^®^). Dogs were induced with propofol (PropoFlo^™^, Zoetis^®^) 2–4 mg kg^-1^ IV at a rate of 1.0 mg kg^-1^ min^-1^ until intubation could be achieved. The dogs were intubated when there was an absence of palpebral reflex, the eye was rotated ventrally, and a lack of jaw tone was evident. Anesthesia was maintained with isoflurane (Isoflurane USP, Akorn) and 100% oxygen; the vaporizer was set at 1.0–1.3% with an oxygen flow rate of 1L min^-1^. The dogs were placed in right lateral recumbency. Lactated Ringer’s (Lactated Ringer’s Injection USP, B. Braun) was administered at 5 ml kg^-1^ hr^-1^ IV. After induction, lidocaine (2.5 mg) was injected SC at dorsal pedal catheter site with an insulin syringe (U-100 Insulin Syringes with Ultra-Fine^™^ needle, BD^®^). Arterial catheter was placed in the dorsal pedal artery using a 22-gauge catheter for invasive blood pressure monitoring. Electrocardiogram, pulse oximetry, body temperature, invasive blood pressure (SAP, MAP, DAP), end-tidal carbon dioxide, and fraction of expired isoflurane were monitored continuously (Datascope Spectrum^®^ and Gas Module 3^™^, Mindray). The dorsal pedal arterial catheter was attached to a low-compliance pressure tubing that connected to electronic pressure transducer positioned and zeroed at the level of the sternal manubrium. Anesthetic depth was assessed as adequate when there was an absence of palpebral reflex, loss of purposeful movement in response to noxious stimulation (toe pinch) with end-tidal isoflurane concentration (Et_ISO_) of 1.0 ± 0.2%. Hypotension was defined as MAP less than 60 mmHg [20,21]. If hypotension occurred prior to maropitant administration, a 5 to 15 ml kg^-1^ fluid bolus of Lactated Ringer’s was administered [22]. If hypotension was accompanied by bradycardia (HR <70 beats per minutes) [23–25] prior to administration of maropitant, glycopyrrolate (0.005–0.01 mg kg^-1^ IV) was administered in conjunction with the fluid bolus. When MAP was maintained above 60 mmHg for at least 5 minutes, maropitant (1 mg kg^-1^) IV was administered over 2 minutes by hand injection using a timer. Heart rate, SAP, DAP, and MAP were recorded before maropitant injection (baseline), at the start of injection (T_0_), during the first 60 seconds of injection (T_a_), during the second 60 seconds of injection (T_b_), at completion of the injection (T_c_) and every 2 minutes post injection (T_-2_) for 18 minutes (T_-18_). Ephedrine (0.1 mg kg^-1^ IV) was the planned rescue protocol for dogs in which MAP was < 50 mmHg for ≥ 5 minutes duration after maropitant injection. All research beagles were returned to ISU LAR at the completion of the study. Recording of the blood pressure and heart rate values was performed by a non-blinded observer.

All variables were compared between groups and from baseline within each group. A Generalized Linear Model with mixed effects was fitted using SAS^®^ software (version 9.4.; SAS^®^ inst. Cary, NC). The fixed effects were Method and Time; the random effects were beagles. All time points were compared to baseline and adjusted using a Bonferroni correction. Differences were considered statistically significant when p ≤ 0.05. The results were expressed as mean ± SD. The sample size was determined using a paired t-test with type I error of 5%, power of 80%, with an effect size of 1.0 based on a previous maropitant study by *Boscan et al* [14].

## Results

**Fig 1:**
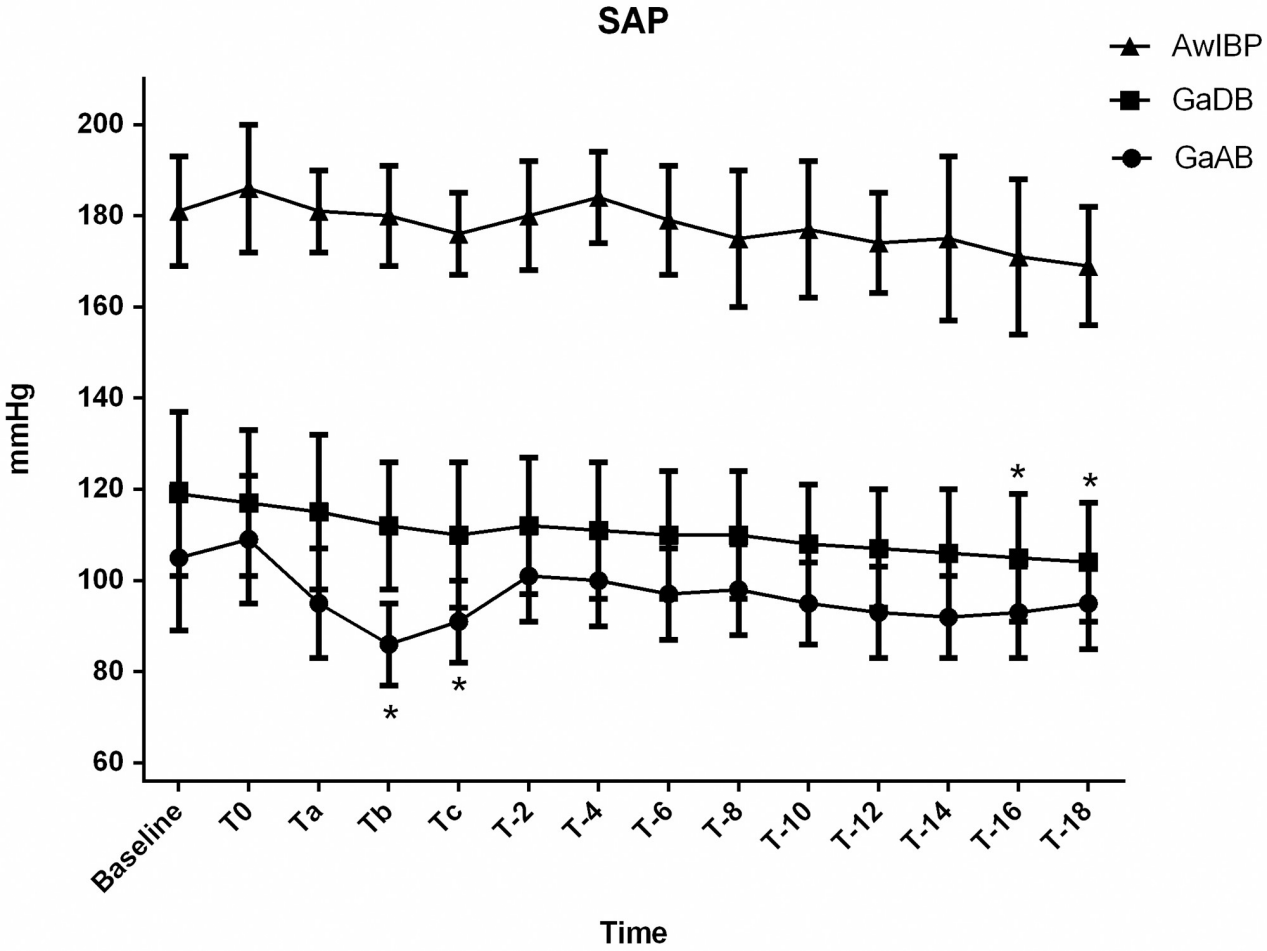
Systolic blood pressure (SAP) of the awake dogs with invasive blood pressure monitoring (AwIBP) and the anesthetized dogs premedicated with acepromazine (GaAB) or dexmedetomidine (GaDB). Values are expressed as mean ± SD. * Denotes value within a treatment group that differs significantly (p≤0.05) from baseline.

**Fig 2:**
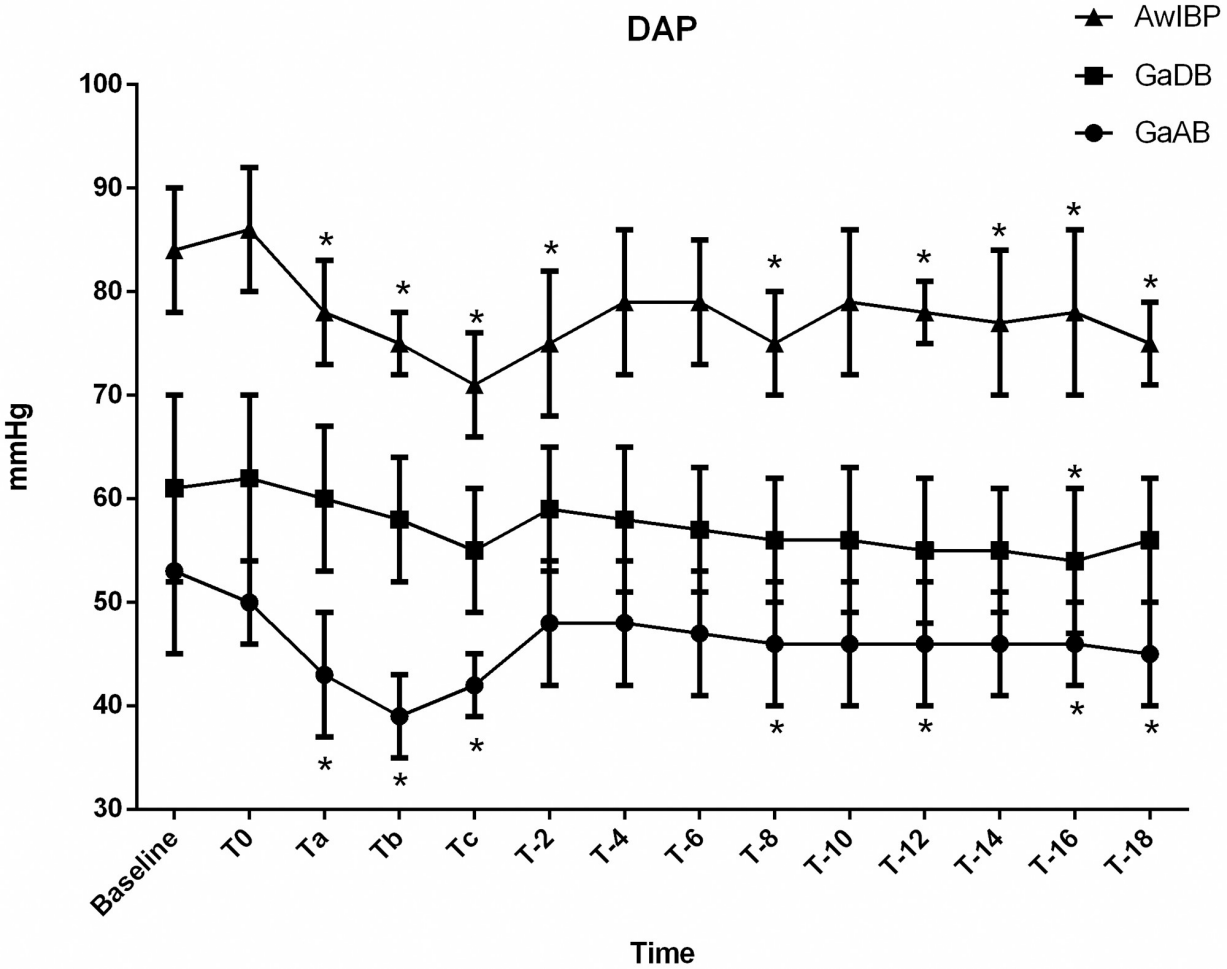
Diastolic blood pressure (DAP) of groups AwIBP, GaAB and GaDB. Values are expressed as mean ± SD. * Denotes value within a treatment group that differs significantly (p≤0.05) from baseline.

**Fig 3:**
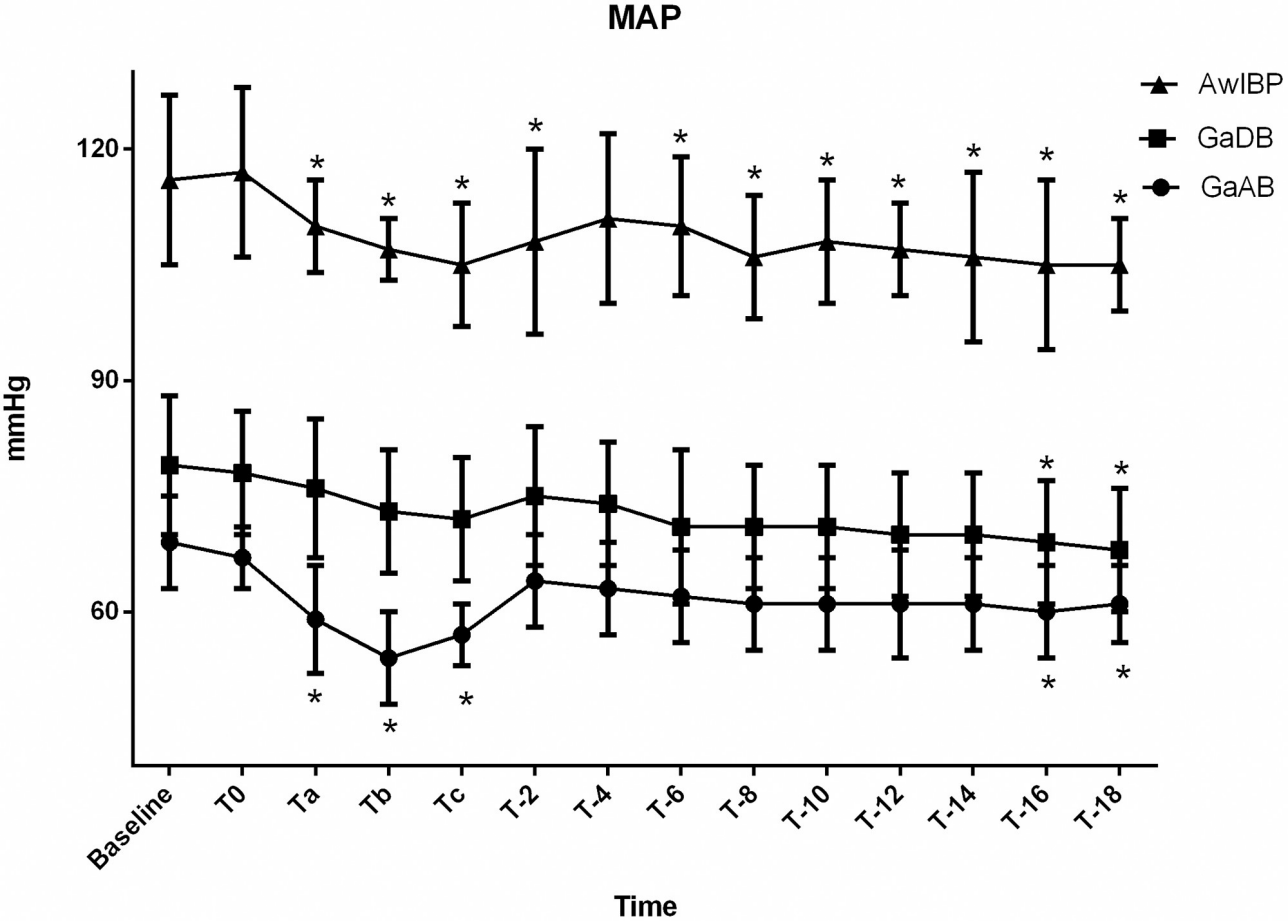
Mean arterial blood pressure (MAP) of groups AwIBP, GaAB and GaDB. Values are expressed as mean ± SD. * Denotes value within a treatment group that differs significantly (p≤0.05) from baseline.

**Fig 4:**
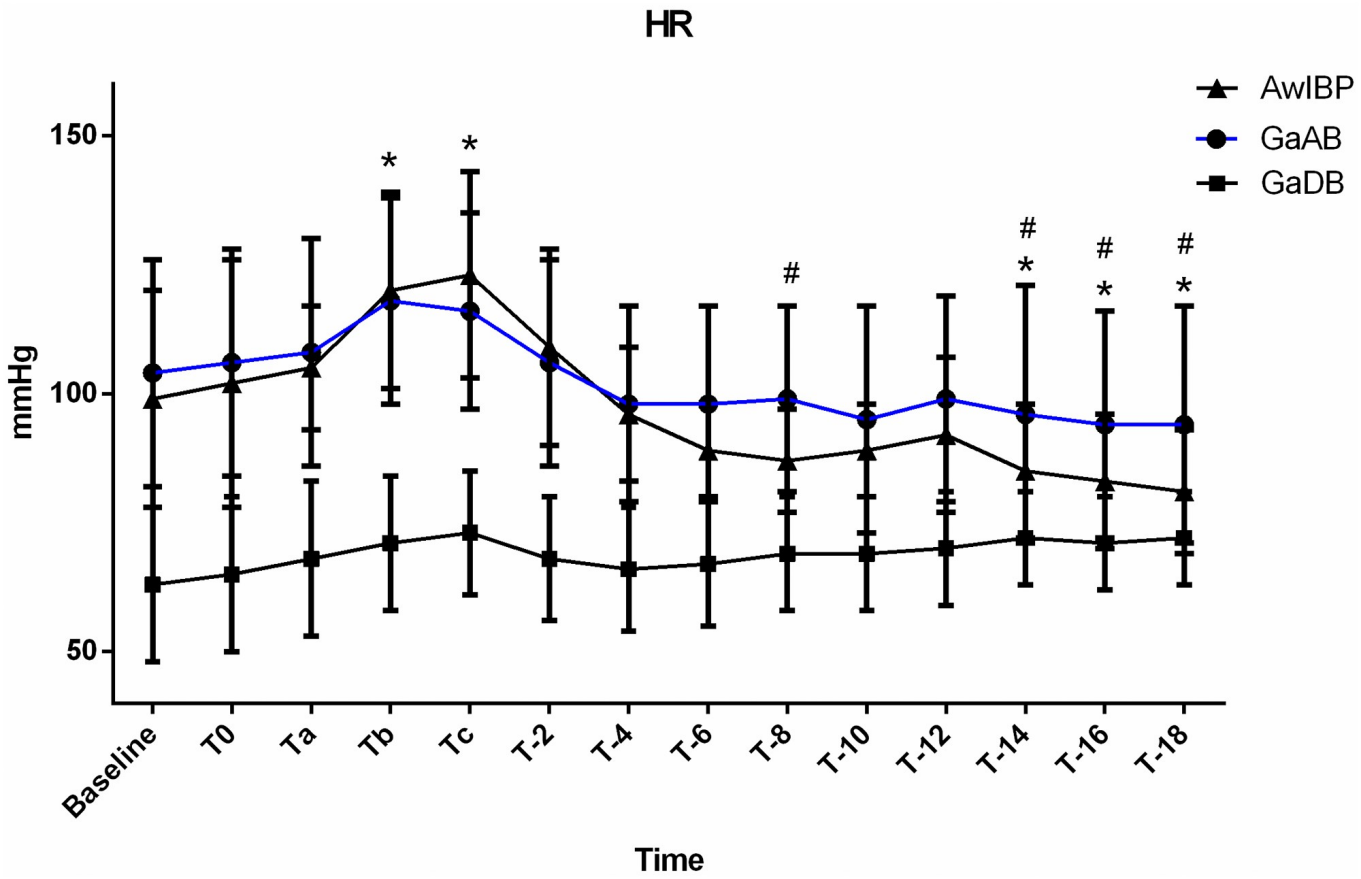
Heart rate (HR) of groups AwIBP, GaAB and GaDB. Values are expressed as mean ± SD. *Denotes value differs significantly (*p* ≤0.05) from baseline value in AwIBP group. # Denotes value differs significantly (*p* ≤0.05) between AwIBP and GaAB groups. There was no significant change in HR within the GaAB and GaDB groups compared to the baseline HR.

Blood pressure and heart rate changes associated with maropitant administration in the awake and anesthetized groups are summarized in Table 1 and Figs 1–4.

**Table 1:** Non-invasive blood pressure values in the awake dogs. Values are expressed as mean ± SD. *p* ≤0.05 denotes statistical significance compared to baseline.

Time	SAP (*p*)	MAP (*p*)	DAP (*p*)
Baseline	160 ± 11	121± 14	99 ± 8
Tc	161 ± 13 (*p* = 1.0)	115 ± 11 (*p* = 1.0)	93 ± 8 (*p* = 0.88)
10 min	159 ± 19 (*p* = 1.0)	125 ± 17 (*p* = 1.0)	97 ± 17 (*p* = 1.0)
20 min	153 ± 16 (*p* = 0.89)	118 ± 15 (*p* = 1.0)	92 ± 15 (*p* = 0.29)

There were no significant changes in blood pressure over time compared to baseline in the awake group in which blood pressure was measured with non-invasive methods (AwNIBP) (*p* > 0.05).

There was significant difference in SAP, DAP, and MAP over time (*p* <0.0001) and between GaAB, GaDB, and AwIBP (*p*<0.0001).

In the awake group with invasive blood pressure monitoring (AwIBP), there was a significant decrease in MAP from the first 60 seconds of injection (T_a_) thru 2 minutes post injection (T_-2_), and again at 6 minutes post injection (T_-6_) thru 18 minutes (T_-18_) compared to baseline. A significant decrease in DAP was also observed from the first 60 seconds of injection (T_a_) to 2 minutes post injection (T_-2_), at 8 minutes post injection (T_-8_), and 12 minutes post injection (T_-12_) thru 18 minutes post injection (T_-18_) compared to baseline.

In the anesthetized dogs, the group premedicated with acepromazine (GaAB) showed a significant decrease in SAP during the second minute of injection (T_b_) and at the completion (T_c_) of injection compared to baseline. A significant decrease in MAP was observed during injection of maropitant (Ta–Tc). This group also had a significant decrease in DAP during injection (Ta–Tc) and at 8 minutes (T_-8_), 12 minutes (T_-12_), and 16 (T_-16_) thru 18 minutes (T_-18_) post injection compared to baseline. In the anesthetized dogs premedicated with dexmedetomidine (GaDB), a significant decrease in SAP, MAP and DAP compared to baseline was only observed at T_-16_. A significant decrease in SAP and MAP was also observed at T_-18_ in the GaDB group.

Clinically significant hypotension (defined as MAP less than 60 mmHg) [20,21] was only observed in the group premedicated with acepromazine (GaAB). Hypotension occurred during injection and immediately post injection with a mean MAP (SD) of 54 ± 6 mmHg during the second minute of injection (T_b_). Prior to injection of maropitant, five of the eight dogs required a fluid bolus and two of the eight dogs also required treatment with glycopyrrolate IV to achieve MAP > 60 mmHg in GaAB group. None of the other groups required treatment of hypotension prior to maropitant administration. No dogs required ephedrine administration during the study.

The awake dogs (AwIBP) were the only group that experienced a significant change in heart rate over time compared to baseline. This group had a significant increase in heart rate during the second minute of injection (T_b_) and at the completion of injection (T_c_) compared to baseline, and significant decrease in heart rate from T_-14_ to T_-18_ compared to baseline. There was a significant difference in heart rate between the awake dogs (AwIBP), and the anesthetized dogs (GaAB, GaDB (*p* <0.0001)) over time. The dogs receiving dexmedetomidine (GaDB) had significantly lower heart rates compared to the awake dogs (AwIBP) and dogs receiving acepromazine (GaAB) (*p* <0.0001) during all time points. Dogs in the awake group (AwIBP) had significantly lower heart rates compared to dogs receiving acepromazine (GaAB) (*p* <0.05) at T_-8_, T_-14_, T_-16_, T_-18_.

## Discussion

This study is the first to evaluate blood pressure and heart rate changes associated with intravenous maropitant administration in awake and anesthetized dogs. Intravenous administration of injectable maropitant causes a significant decrease in blood pressure during injection and immediately post injection in awake dogs and in anesthetized dogs premedicated with acepromazine. The change in blood pressure was significant over time in each of the individual groups.

Dogs that were premedicated with acepromazine and anesthetized with isoflurane exhibited clinical hypotension even prior to maropitant administration. Five out of eight dogs required treatment to become normotensive. Published studies have reported that the hypotensive effects of acepromazine are enhanced by the decrease in systemic vascular resistance associated with isoflurane [18,26]. In the present study, hypotension caused by acepromazine and isoflurane was treated with crystalloid fluid boluses. Hypotension caused by absolute hypovolemia, such as dehydration or hemorrhage, is usually responsive to fluid therapy [27,28]. However, hypotension that is caused by vasodilation (relative hypovolemia) from anesthetic drugs has an unpredictable responsiveness to fluid therapy [29,30]. A fluid challenge with crystalloid fluids is recommended in an effort to assess fluid responsiveness in hypotensive dogs under general anesthesia [28,31–33]. For this study, fluid treatment for hypotension followed the American Animal Hospital Association (AAHA) Guidelines for fluid therapy for dogs and cats [22]. Hypotension that occurred with concurrent bradycardia was treated with glycopyrrolate. All dogs were able to maintain normotension for 5 minutes prior to maropitant administration.

Clinically significant arterial hypotension was observed without a significant compensatory increase in heart rate during maropitant injection in the acepromazine group. Conversely, the awake group had a significant increase in HR during the period of lower MAP and DAP associated with maropitant injection. Inhibition of sympathetic activity, adrenergic neurotransmission, and baroreceptor reflex sensitivity by the inhalant and phenothiazine, are possible factors that contributed to a depressed compensatory response to hypotension during maropitant injection [28]. Persistent blood pressure reduction that required treatment with ephedrine was documented in a study that evaluated the antinociceptive effects of maropitant (5.0 mg kg^-1^ IV) in cats receiving an IV bolus of maropitant with morphine and acepromazine as premedication [13]. Our study used the lower label dose of maropitant (1 mg kg^-1^) which resulted in transient hypotension with a return to normotension without requiring rescue blood pressure treatment with ephedrine. Intravenous administration of maropitant may have a dose dependent response on blood pressure and/or potential species differences in the severity of blood pressure effects.

The dexmedetomidine group did not experience a significant decrease in blood pressure associated with maropitant injection. Dexmedetomidine is an α_2_-receptor agonist that produces a biphasic hemodynamic response. Activation of subtype α_2_B-receptorsin the vascular smooth muscle leads to vasoconstriction and decreased heart rate (phase 1). As dexmedetomidine plasma concentration decreases, vasodilation occurs due to α_2_A-receptor activation in vascular endothelium and central effects from decreased norepinephrine release [34,35]. In dogs, decreased blood pressure during phase 2 is associated with bradycardia from central sympatholytic effects with peripheral effects of α_2_-agonist subsiding [36,37]. The duration of effect of dexmedetomidine is approximately 1 hour or less depending on dose [37]. The time from premedication to the time prior to maropitant injection was approximately 30 to 40 minutes in this study. The decrease in blood pressure in the dexmedetomidine group that was observed at 16 to 18 minutes post injection may be due to the sympatholytic effect of the dexmedetomidine and not necessarily associated with maropitant. Dexmedetomidine may be able to attenuate the vasodilatory effect of maropitant during the injection since there is no decrease in blood pressure during injection.

The mechanism of blood pressure reduction during maropitant administration remains unclear, but may be associated with the formulation of injectable maropitant which contains maropitant, sulfobutylether-beta-cyclodextrin, and metacresol [38]. Sulfobutylether-beta-cyclodextrin is a cyclic oligosaccharide that acts as a carrier molecule for maropitant. Presently, there is no evidence that the dose of cyclodextrin in injectable maropitant has a significant effect on mean arterial pressure or heart rate with intravenous injection in anesthetized dogs [39,40].

The preservative, metacresol, is a common excipient in insulin formulations for humans. Currently, there is no literature that documents the blood pressure effects of metacresol injection in veterinary medicine. Reported side effects of metacresol in human medicine include localized allergic skin reactions, l cytotoxicity to fibroblast cell, human adipocyte, and monocytic cells [41–43]. Further investigation is needed to evaluate the effect of metacresol on blood pressure. Another formulation of injectable maropitant (Prevomax^®^, Dechra^®^) is available which does not contain metacresol. This formulation is marketed in Europe for veterinary use and is currently not available in the United States. In the European formulation, the excipient contains benzyl alcohol, betadex sulfobutyl ether sodium, citric acid anhydrous and sodium hydroxide [44]. There is currently no information regarding the effects of IV administration of this formulation on blood pressure and future studies are warranted.

Neurokinin receptors are associated with blood pressure regulation [45,46]. Neurokinin-1 agonists can cause centrally mediated vasoconstriction or peripheral vasodilation. The NK-1 agonist, SP, activates tachykinin NK-1 receptors on the vascular endothelium to cause release of endothelial nitric oxide promoting vascular smooth muscle relaxation [47]. Spinal cord and intracerebroventricular activation of NK-1 receptors by substance P in rats evokes a vasopressor and positive chronotropic response, peripheral activation caused a vasodilatory effect [45,48]. Studies in dogs have found that SP has vasodilatory effects on hepatic arterial and portal vascular beds [49].

In human clinical trials, both aprepitant and fosaprepitant, which are NK-1 antagonists used for oral/intranasal and IV administration, respectively, are reported to be associated with a decrease in blood pressure or the occurrence of hypotension [50–53]. Maropitant may interact with NK-1 receptors centrally and peripherally causing transient decreases of blood pressure and a reflex chronotropic response in dogs as we observed in this study.

Arterial blood pressure is regulated by the autonomic nervous system, renin angiotensin system, and the arginine vasopressin pressor systems in dogs [54,55]. The significant increase in HR observed in the awake dogs may have been due to activation of the autonomic nervous system to compensate for the decrease in blood pressure during maropitant injection. The underlying mechanism is unknown but may be associated with modulation of the cardiovascular system and sympathetic system by central NK-1 receptors [56].

The non-invasive blood pressure measurement method was unable to detect the effect of blood pressure changes over time. Non-invasive blood pressure measurement in awake patients requires a minimum of three to five consecutive values and the values also need to have less than 20% variability to be considered accurate [57,58]. Our data was collected with less than 20% variability and the readings were taken at 5 minutes intervals. Oscillometric blood pressure was unable to identify real-time pressure changes that were detected with invasive blood pressure measurement. Blood pressure changes caused by maropitant during intravenous administration potentially may not be observable with oscillometric methods of blood pressure monitoring due to these limitations.

Results of this study only hold true for the drug doses that were used in this study. Drug doses were chosen based on doses used for premedication on canine anesthesia patients at this institution. At our institution, 0.005 mg kg^-1^ acepromazine is a standard dose when administered concurrently with an opioid analgesic drug such as butorphanol. The choice of drug doses for dexmedetomidine and butorphanol falls within published dosing ranges [59]. The acepromazine dose that was used in this study was lower than the referenced dosing range [59]. A published retrospective study cited a clinical dosing of 0.005 to 0.07 mg kg^-1^ of acepromazine for preanesthetic medication or tranquilization [60]. Published label drug doses are for administration of the single agent. Multi-modal premedication dictates that when using multiple drugs, the dose of each individual drug is decreased in order to take advantage of the desired attributes of each drug but limit the unwanted side effects of each drug.

The goal of subcutaneous lidocaine injection prior to intravenous and intraarterial catheter placement was to decrease discomfort and pain associated with catheterization. Pain from local anesthetic injections primarily occurs with needle insertion and infiltration of the drug [61]. To minimize injection pain associated with lidocaine, subcutaneous injection of lidocaine with 30-gauge needle was used. Smaller needle size and subcutaneous injection instead of intradermal injection have proven to decrease injection pain with local anesthetic [61,62]. In awake dogs, response to pain, such as vocalization or increased heart rate and blood pressure, was absent during lidocaine injection for catheter placement. The effect of administration of lidocaine for catheter placement was assumed to be minimal.

One limitation of this study was that a control group with dogs anesthetized with only isoflurane without premedication was not included. Previous studies have already suggested that a decrease in blood pressure was associated with IV maropitant injection in dogs anesthetized with inhalant anesthesia without premedication [10,14]. Not including such a control group was due to considerations for laboratory animal welfare. Administration of appropriate premedication prior to inhalant anesthesia minimizes patient discomfort and stress and also reflects recommendations put forth by the American Animal Hospital Association (AAHA) guidelines for anesthesia [63]. Lack of blinding of the observer was another limitation. Since recording of the data in this study was an objective value rather than a subjective value, the influence of non-blinding on data recording is assumed to be minimal.

An additional limitation was that cardiac output was not measured precluding differentiation of cardiac output changes versus systemic vascular resistance as responsible for the blood pressure changes associated with maropitant administration. We also did not measure invasive blood pressure and non-invasive blood pressure concurrently in each group. This was due to the inability to collect all five necessary consecutive readings within two minutes due to the natural limitations of the oscillometric blood pressure device used in the study. The oscillometric device we used will take more than 2 minutes to cycle for five blood pressure readings. We did not investigate the excipients of the maropitant formulation. We cannot isolate the effects of maropitant from that of the carrier formulation. However, current literature in humans suggests that blood pressure changes occur with different formulations of NK-1 antagonists, indicating that the blood pressure effects observed are most likely associated with the NK-1 antagonist themselves and not the excipient. Lastly, healthy research beagles with intact cardiovascular responses were used in this study, therefore, results may not be extrapolated to critically ill patients or patients with cardiovascular compromise.

## Conclusion

Intravenous administration of maropitant is associated with a transient decrease in blood pressure in awake dogs with a concomitant increase in HR. Clinically significant hypotension occurred in dogs premedicated with acepromazine, induced with propofol, and maintained on isoflurane. Dogs premedicated with dexmedetomidine did not experienced a significant decrease in blood pressure associated with IV maropitant administration during isoflurane anesthesia. Maropitant should be administered slowly when given IV and blood pressure and heart rate should be monitored during and after administration, especially in anesthetized dogs receiving acepromazine premedication.
